# Enhancing Fracture Toughness of Dental Zirconia through Incorporation of Nb into the Surface

**DOI:** 10.3390/ma17184446

**Published:** 2024-09-10

**Authors:** Seiji Ban, Yuta Yasuoka

**Affiliations:** 1 Department of Dental Materials Science, School of Dentistry, Aichi Gakuin University, Nagoya 464-8650, Japan; 2 KCM Corporation, Nagoya 455-8668, Japan; yasuoka_yuta@kyoritsu-kcm.co.jp

**Keywords:** zirconia, fracture toughness, niobium, surface modification, thermal expansion

## Abstract

Background: Our previous study found that the addition of pentavalent cations like niobium (Nb) to yttria-stabilized zirconia increased fracture toughness but also raised the coefficient of thermal expansion (CTE), and opacity also increased undesirably. A new surface treatment is required to boost fracture toughness without altering CTE or translucency. Methods: The surfaces of pre-sintered 3 mol% yttria-stabilized tetragonal zirconia polycrystal (3Y-TZP) and 4.2 mol% yttria-stabilized partially stabilized zirconia (4.2Y-PSZ) were treated with a Nb sol solution containing Nb_2_O_5_ nanoparticles. After drying and sintering, a high-Nb-content surface layer formed with a depth of approximately 1 mm. Results: The Nb content in this surface layer matched that of a bulk material with 1 mol% Nb_2_O_5_. The tetragonality of the surface zirconia increased, enhancing the surface fracture toughness without changing the CTE or translucency. Conclusions: Adding Nb near the surface improved the fracture toughness without affecting the CTE or translucency. This method could strengthen zirconia prostheses, allowing more reliable dental restorations.

## 1. Introduction

Dental zirconia has evolved significantly and is now widely used as an aesthetic dental restorative material. Yttria (Y_2_O_3_) is commonly used as a stabilizer to enhance strength and toughness. When the yttria content ranges from 3 to 8 mol%, both the tetragonal and cubic phases coexist at room temperature, forming partially stabilized zirconia (PSZ). At around 3 mol%, the tetragonal phases approach 100% at room temperature, forming tetragonal zirconia polycrystal (TZP). Initially, 3 mol% yttria-stabilized TZP (3Y–TZP) was introduced as a core material to replace metal in dental applications. However, due to its low translucency, the surface of 3Y-TZP must be veneered with feldspathic porcelain to enhance its appearance. To exploit the high strength of zirconia without porcelain veneering, high-translucency 4 mol% and 5 mol% yttria-stabilized PSZ (4Y–PSZ and 5Y–PSZ) have been developed for monolithic zirconia prostheses [1]. Increasing the translucency of PSZ can be achieved by increasing the stabilizer content, such as yttria, though this may reduce the strength of zirconia [2]. Additionally, ultra-high-translucency zirconia (UHTZ) has been developed using novel manufacturing techniques to achieve particularly high translucency [3]. Consequently, numerous types of zirconia are available for dental treatment, classified mainly into three categories: single colors with different yttria contents, single-composition multilayer types (uniform composition with different colored multilayers), and mixed-composition multilayer types (multilayers with different compositions and coloring). Based on the yttria content and multilayer construction, these can be classified into 13 types.

In our previous study, forty-four types of zirconia were prepared by combining yttrium (Y), ytterbium (Yb), niobium (Nb), and tantalum (Ta) oxide as stabilizers and were evaluated for fracture toughness and opacity [4]. The results indicated that adding trivalent cations Y and/or Yb reduced fracture toughness and opacity, whereas adding pentavalent cations Nb and/or Ta to ZrO_2_ stabilized with trivalent cations increased both properties. There was no significant difference in the effects between Y and Yb or between Nb and Ta. Furthermore, these effects were similar for additions to 3Y–TZP and 4Y–PSZ.

However, while the addition of pentavalent cations increased the fracture toughness of zirconia, it also increased the coefficient of thermal expansion (CTE) and the opacity. As the opacity increases, indicating decreases in translucency, it becomes necessary to improve aesthetics by applying porcelain veneering or glazing to the surface. Veneer porcelains and glazing materials for zirconia are designed to match the original CTE of zirconia, and changes in CTE can render these coatings unusable. Therefore, maintaining a constant CTE and translucency is crucial. In this study, we developed a novel surface treatment using a Nb sol for zirconia to enhance fracture toughness while sustaining the original CTE and translucency. This approach utilizes a novel technique to address the inherent weaknesses of zirconia, particularly at stress concentration points, thereby producing highly reliable dental restorations.

## 2. Materials and Methods

### 2.1. Preparation

Table 1 displays the chemical composition of four zirconia powders prepared by combining yttrium and niobium oxide as stabilizers and two Nb sol specimens.

**Figure 1:**
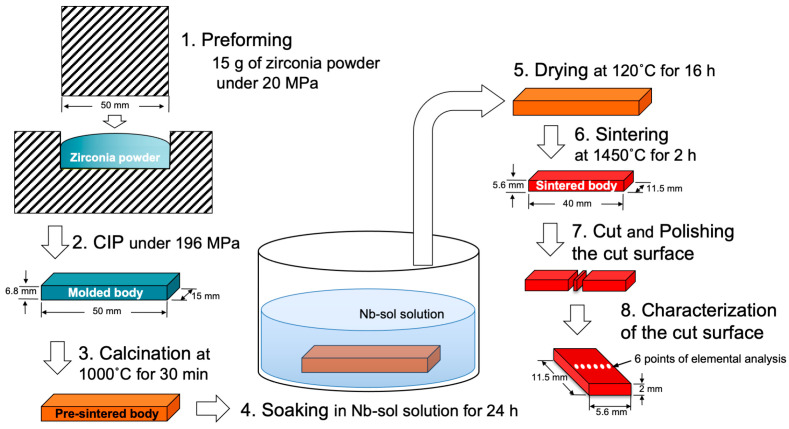
Schematic illustration of the specimen preparation process, excluding thermal expansion and opacity. The preparation methods for specimens used in the thermal expansion and opacity studies were nearly identical, with variations only in specimen size, shape, powder weight, pressing conditions, and soaking time.

Zirconia powders (KCM Corporation, Nagoya, Japan) containing 3 mol% and 4.2 mol% yttrium oxide (Y_2_O_3_) (3Y–TZP and 4.2Y–PSZ) were prepared following our previous method [4]. Additionally, two materials (3Y–1 mol% Nb_2_O_5_ and 4.2Y–1 mol% Nb_2_O_5_) were prepared by mixing Nb_2_O_5_ with 3Y–TZP and 4.2Y–PSZ powders to achieve a 1 mol% Nb_2_O_5_ concentration in the entire mixture. Figure 1 shows the specimen preparation process, excluding thermal expansion and opacity. After mixing, 15 g of these powders was placed into a mold with a bottom surface area of 50 mm by 15 mm, and a preformed body was created under a pressure of 20 MPa.

For thermal expansion and opacity specimen preparation, 1.5 g and 1.7 g of these powders were filled into molds with diameters of 7 mm and 20 mm, respectively, and subjected to a pressure of 0.78 MPa to produce preformed bodies. After removing these preforms from the molds, they were subjected to CIP molding at a pressure of 196 MPa to produce molded bodies. These molded bodies were calcined at 1000 °C for 30 min to obtain pre-sintered bodies. The pre-sintered bodies were then fired at 1450 °C for 2 h to obtain sintered bodies.

The sintered body intended for general measurement had a rectangular shape with dimensions of 40 mm in length, 11.5 mm in width, and 10 mm in height. The sintered body for thermal expansion measurements had a cylindrical shape with a diameter of 5.5 mm and a height of 10 mm. The sintered body for opacity measurements had a disc shape with a diameter of 14.5 mm and a thickness of 1.6 mm, and the thickness was adjusted to 1.5 mm by mirror polishing.

### 2.2. Nb Incorporation into Zirconia Surface

For the preparation of 3Y- and 4.2Y-Nb sol, the pre-sintered bodies of 3Y–TZP and 4.2Y–PSZ were soaked in Nb_2_O_5_ sol (Biral Nb–G6000, Taki Chemical Co., Ltd., Kakogawa, Hyogo, Japan) for 24 h for the specimens other than those for thermal expansion, and for 4.5 h for thermal expansion specimen, then dried at 120 °C for 16 h.

After drying, the Nb-incorporated pre-sintered specimens were sintered at 1450 °C for 2 h to obtain the final sintered body. The Nb-incorporated sintered body was cut perpendicular to its long side (40 mm side) to prepare specimens for fracture toughness and Nb concentration analysis, with a cut surface measuring 11.5 mm by 5.6 mm at the midpoint of the long side using a diamond cutter. The cut surface was mirror-polished with a diamond paste.

### 2.3. Fracture Toughness

The indentation fracture (IF) method was employed to determine the depth profile of fracture toughness in this study. According to the IF method specified in JIS R 1607:2015, “Testing methods for fracture toughness of fine ceramics at room temperature”, fracture toughness values were determined using a Vickers hardness tester (MV–1, Matsuzawa Co., Ltd., Akita, Japan) for each specimen according to the following equation:

*K*_1c_ = 0.18(*E*/*H*v)^0.5^(*P*/*c*^1.5^), (1)

where *K*_1C_ is the fracture toughness (Pa√m), *E* is the modulus of elasticity (Pa) (206 GPa was employed in this study), *H*_V_ is the Vickers hardness (Pa) [*H*v = 0.1891*P*/(2*a*)^2^], *P* is the indentation load (N) (98 N was employed in this study), *c* is half of the average crack length (m), and *a* is half of the average diagonal length of the indenter (m). Measurements were performed on the surface of six types of sintered bodies and the section of the 3Y-Nb sol sintered body. Five measurements were made per specimen.

### 2.4. Thermal Expansion

The average linear expansion coefficient from 25 °C to 500 °C was measured using a thermomechanical analyzer (TMA4000SA, Netzsch-Geratebau GmbH, Selb, Bayern, Germany). The measurement was performed according to JIS R 1618 under the following conditions: a load of 20 g, a heating rate of 5 K/min, and air atmosphere.

### 2.5. Opacity

Using a spectrophotometer (CM-3700d, Konica Minolta Inc., Tokyo, Japan), the reflectance (*R*_1_ and *R*_0_) of a disc-shaped specimen with a thickness of 1.5 mm was measured against a black and white backgrounds, separately. The opacity (%) was automatically calculated from the measurement results according to the formula:

Opacity (%) = *R*_1_/*R*_0_ × 100, (2)

when measuring the reflectance from 360 to 740 nm, a calibration plate (CM–A90) and a zero-calibration box (CM-A94) were used as white and black backgrounds, respectively. The measurement geometry was set to SCI reflectance, with an 8 mm measurement diameter, 100% full UV condition, 10° viewing angle, and D65 light source. Measurements were repeated 3 times for each specimen, and the average value over the 3 measurements was calculated.

### 2.6. Nb Concentration Analysis

After mirror-finishing the cut surface of the 3Y-Nb sol, the cut surface was analyzed using a scanning electron microscope with wavelength-dispersive X-ray spectroscopy (SEM-WDX) (JXA-iHP200F Hyper Probe, JEOL Ltd., Tokyo, Japan). Two methods were used to measure the Nb concentration’s distribution: a method using the results of the Nb characteristic X-ray intensity from the elemental mapping image and a method using quantitative analysis results.

For Nb elemental mapping, a WDS PET detector was used under the following conditions: accelerating voltage of 15.0 kV, irradiation current of 5.0 × 10^−8^ A, collection time of 10 msec, and pixel size of 2.5 μm (at 100× magnification). Elemental mapping was performed along the short side direction of the cut surface, passing through the center. Six mapping images of Nb concentration and six secondary electron images (SEIs) were combined into a single image of the total cut surface.

Quantitative analysis of Nb in the 3Y-Nb sol sintered body and the 3Y-1 mol% Nb_2_O_5_ was conducted at positions of 0 mm, ±0.5 mm, ±1.5 mm, ±2.5 mm, and ±2.7 mm from the center. This analysis was performed using a Nb WDS PET detector, with an accelerating voltage of 15.0 kV, an irradiation current of 5.0 × 10^−8^ A, and a collection time of 500 msec. The obtained Nb analytical value was normalized by subtracting the background and using the average Nb analytical value of 3Y–1 mol% Nb_2_O_5_ as 1 mol% Nb_2_O_5_.

### 2.7. X-ray Diffractometry

X-ray diffraction (XRD) patterns of the polished specimens were measured using an X-ray diffractometer (Ultima IV, Rigaku Corporation, Tokyo, Japan) under conditions of at 40 kV and 40 mA. Scans were conducted between 72° and 76° in 2*θ* at 0.04°/min using Cu Ka radiation. The lattice constants of the tetragonal *c*-axis and *a*-axis were derived from the (004) and (400) peak positions around 73° and 74.5°, respectively, using analysis software (PDXL2). The tetragonal *c*/*a* axis lattice constant ratios were calculated from these values.

## 3. Results

### 3.1. Fracture Toughness, Thermal Expansion, and Opacity

**Figure 2:**
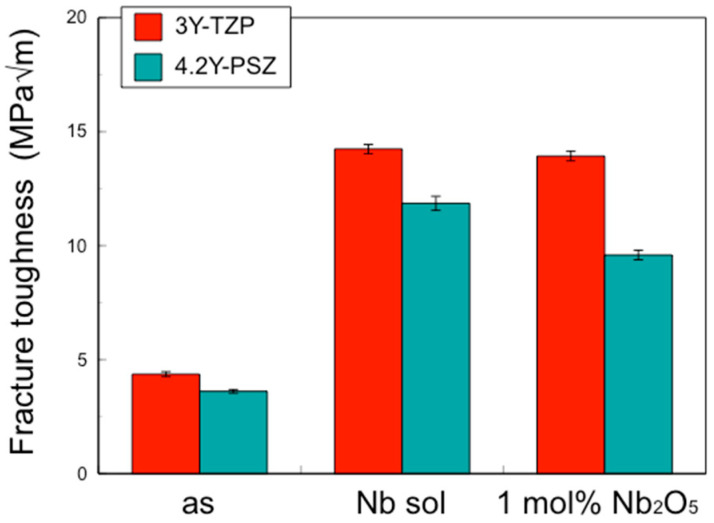
Fracture toughness of the surface of 3Y–TZP, 3Y–Nb sol, 3Y–1 mol% Nb_2_O_5_, 4.2Y–PSZ, 4.2Y–Nb sol, and 4.2Y–1 mol% Nb_2_O_5_.

The fracture toughness of the sintered bodies of 3Y–TZP and 4.2Y–PSZ coated with Nb sol and containing 1 mol% Nb_2_O_5_ was higher than that of= those without Nb_2_O_5_ (Figure 2).

**Figure 3:**
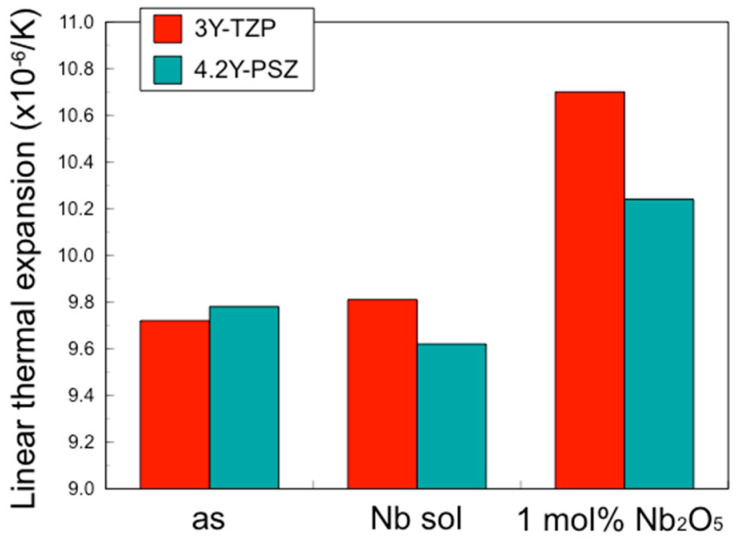
Coefficient of linear thermal expansion of 3Y–TZP, 3Y–Nb sol, 3Y–1 mol% Nb_2_O_5_, 4.2Y–PSZ, 4.2Y–Nb sol, and 4.2Y–1 mol% Nb_2_O_5_.

**Figure 4:**
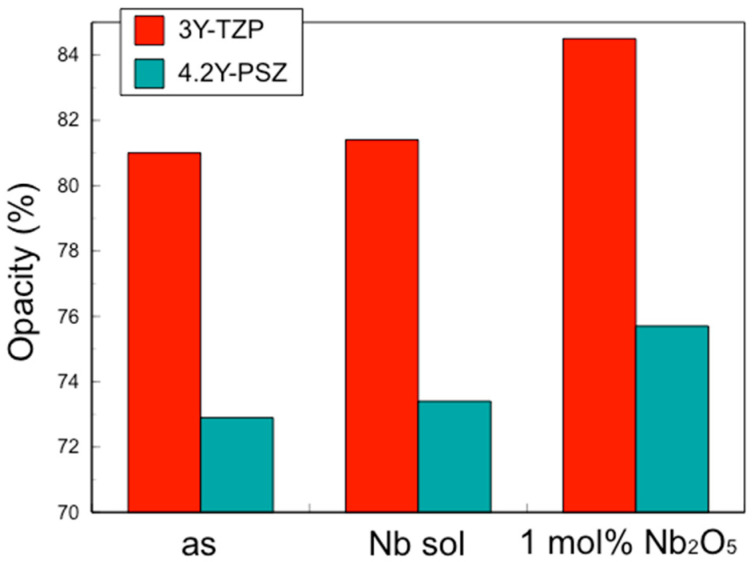
Coefficient of opacity of 3Y–TZP, 3Y–Nb sol, 3Y–1 mol% Nb_2_O_5_, 4.2Y–PSZ, 4.2Y–Nb sol, and 4.2Y–1 mol% Nb_2_O_5_.

Conversely, the coefficient of thermal expansion (CTE) and the opacity of 3Y–TZP and 4.2Y–PSZ containing 1 mol% Nb_2_O_5_ were larger than those without Nb_2_O_5_, while the CTE and the opacity of 3Y–TZP and 4.2Y–PSZ coated with Nb sol were nearly the same as those without Nb_2_O_5_ (Figure 3 and Figure 4).

These results suggest that the incorporation of Nb into the zirconia surface enhanced fracture toughness to the same level as zirconia containing 1 mol% Nb uniformly, while suppressing thermal expansion and opacity.

### 3.2. Distribution of Nb

**Figure 5:**
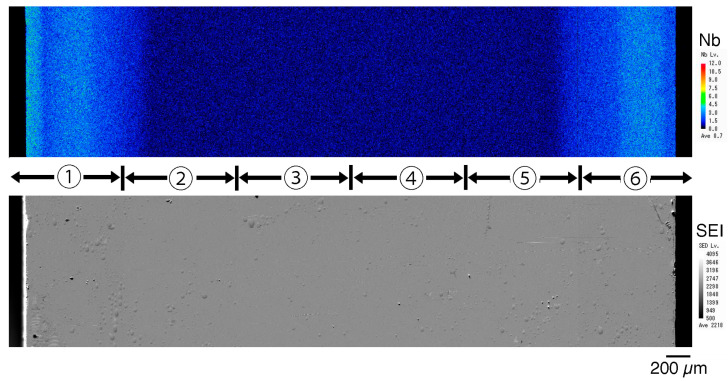
Nb concentration map (**upper**) and SEI (**lower**) of the section of sintered 3Y-Nb sol. Six mapping images of Nb concentration and six SEIs were united into each image of the total cut surface. The relatively bright area indicates high Nb concentration, and the relatively dark area indicates low Nb concentration. The six images obtained for each area numbered in the figure were combined into one image.

In the Nb mapping image of the sintered 3Y–Nb sol section (Figure 5), relatively bright areas indicating high Nb concentration were observed on both sides at an about 1 mm depth from the surface, and relatively dark areas indicating low Nb concentration were observed in the middle of the section.

**Figure 6:**
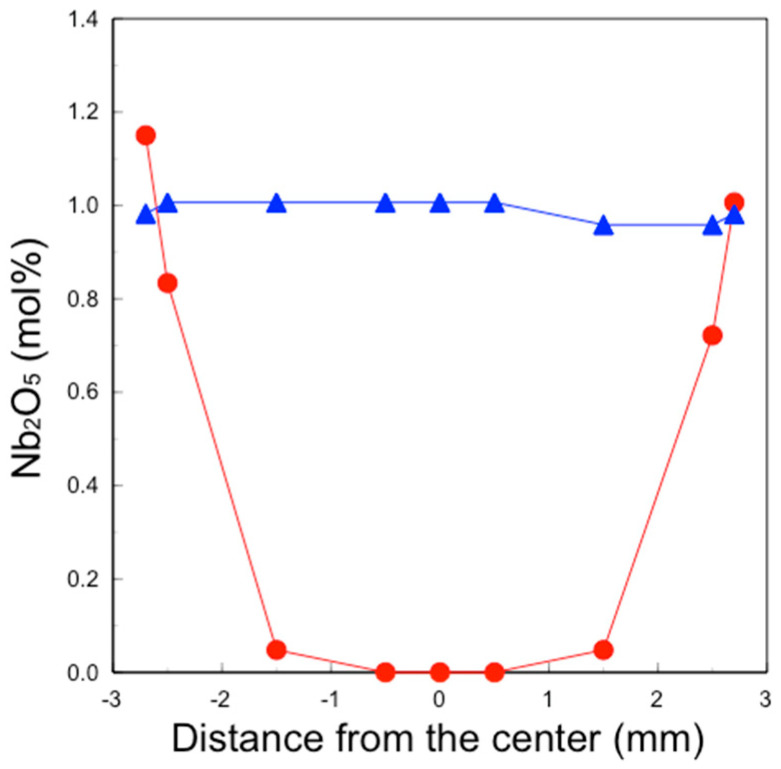
Analyzed Nb_2_O_5_ content of the section of sintered 3Y–1 mol% Nb_2_O_5_ (▲) and sintered 3Y–Nb sol (●). The horizontal axis indicates the distance from the center of the section, and the vertical axis indicates the converted Nb_2_O_5_ concentration.

The qualitative analysis results of Nb_2_O_5_ (Figure 6) showed that the Nb_2_O_5_ concentration in the 3Y–1 mol% Nb_2_O_5_ section was consistently about 1 mol% in all areas, whereas, in the 3Y–Nb sol section, the Nb_2_O_5_ concentration dramatically varied with the distance from the surface. Almost no Nb_2_O_5_ was detected in the middle area, with Nb_2_O_5_ segregated on the surface layer at a depth of 1 mm.

About 1 mol% Nb_2_O_5_ was observed in the outermost surface area. 4.2Y–Nb sol showed a similar Nb distribution to that of 3Y–Nb sol, as not shown in figure.

### 3.3. Change in Crystal Lattice

**Figure 7:**
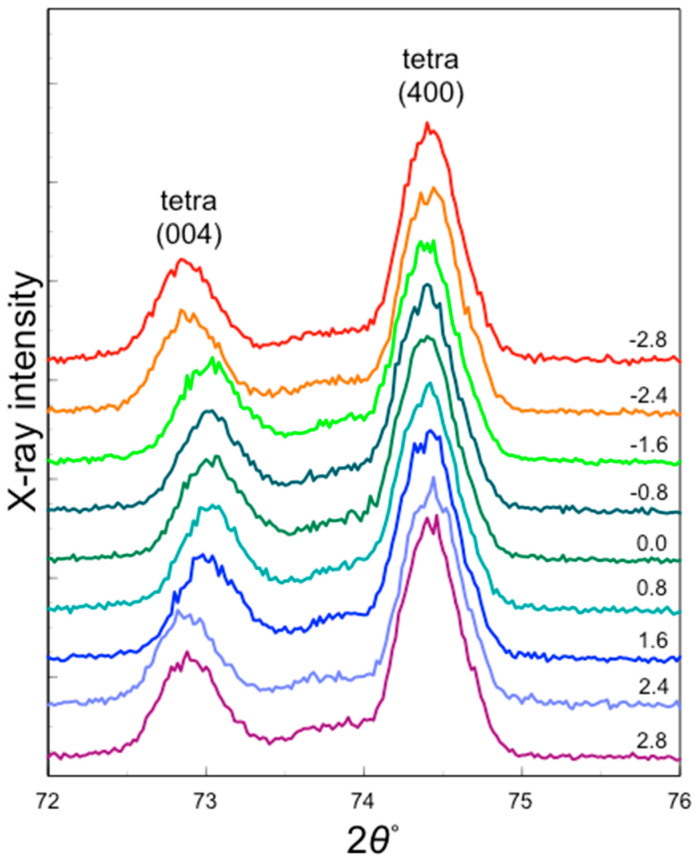
XRD patterns of the section of sintered 3Y–Nb sol. The figures in right end stand for the measurement region distance from the center.

The XRD patterns of the sintered 3Y–Nb sol section varied with the distance from the center (Figure 7).

The diffraction peak around 74.3° in 2*θ* is assigned to (400) of tetragonal zirconia and the peak at 72.8–73.0° in 2*θ* is assigned to (004) of tetragonal zirconia. The peak position of (400) was consistent, whereas the peak position of (004) changed with the distance from the center. The peak positions at −1.6 to 1.6 mm from the center were around 73° in 2*θ*, while the peak position at ±2.4 and ±2.8 mm from the center, indicating the vicinity of the surface, were around 72.8° in 2*θ*.

**Figure 8:**
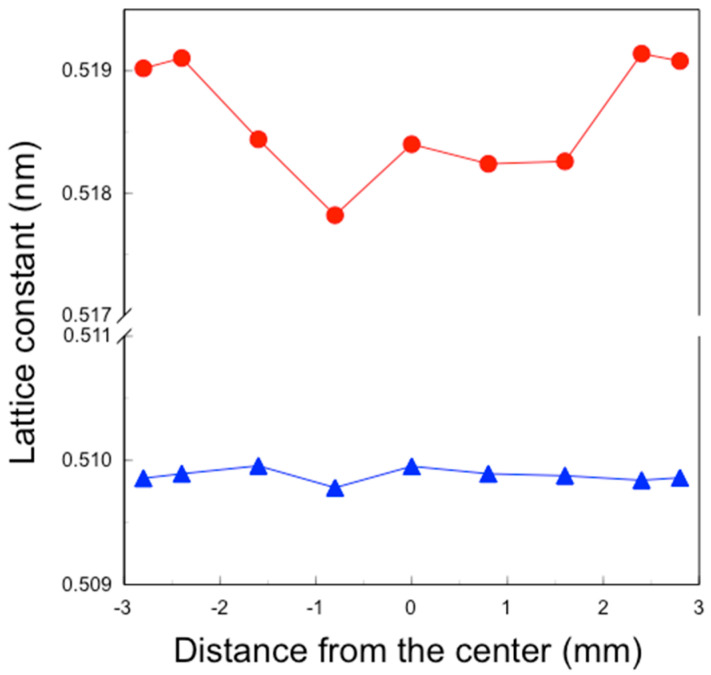
Distribution of lattice constant of *a*-(▲) and *c*-axis (●) of the section of sintered 3Y–Nb sol.

The lattice constants of the *a*- and *c*-axes of the sintered 3Y–Nb sol section were derived from the (400) and (004) peak positions in their XRD patterns (Figure 8).

The lattice constants of the *a*-axis were almost constant at 0.510 nm, whereas those of the *c*-axis varied with the distance from the center. The lattice constants of the *c*-axis at −1.6 to 1.6 mm from the center were about 0.518 nm, while those at ±2.4 and ±2.8 mm from the center were about 0.519 nm.

**Figure 9:**
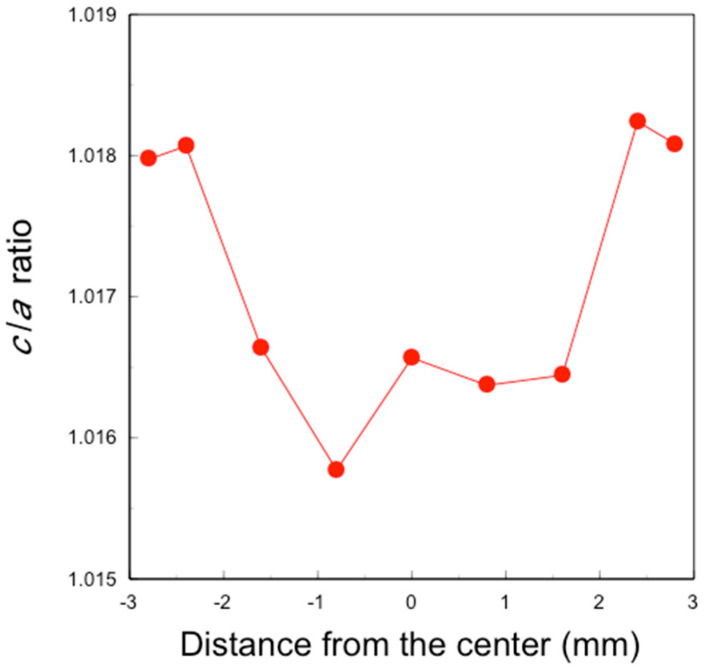
Distribution of lattice constant ratio *c*/*a* of a section of sintered 3Y–Nb sol.

Lattice constant ratios (*c*/*a*) of the sintered 3Y-Nb sol section were derived from these data (Figure 9). The tetragonal *c*/*a* ratio, indicating the stability and the crystallinity of the tetragonal zirconia, was about 1.0158–1.0165 at −1.6 to 1.6 mm from the center, and about 1.018 at ±2.4 and ±2.8 mm from the center.

### 3.4. Distribution of Fracture Toughness

**Figure 10:**
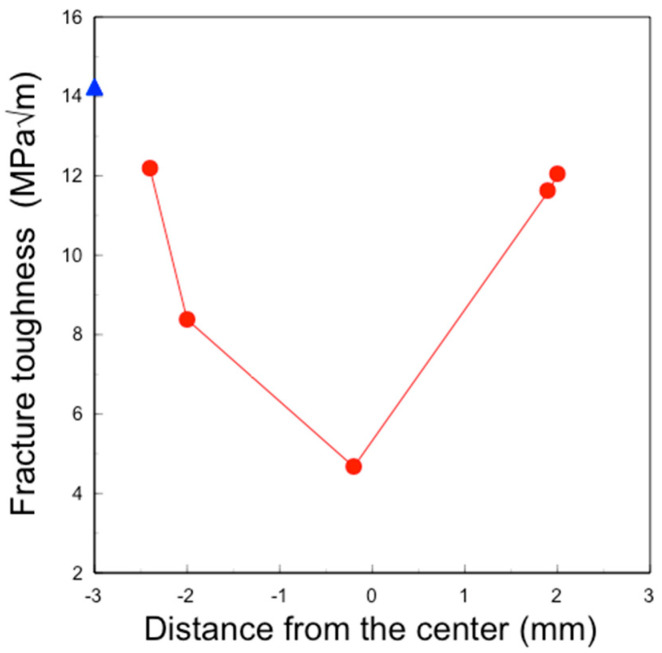
Distribution of fracture toughness of the surface (▲) and the section (●) of sintered 3Y–Nb sol.

The fracture toughness of the sintered 3Y–Nb sol section varied with the distance from the center (Figure 10).

The fracture toughness at the center showed the minimum value of 5 MPa√m, increasing to about 12 MPa√m closer to the surface. The fracture toughness on the surface was approximately 14.2 MPa√m.

## 4. Discussion

Our previous study revealed that the addition of pentavalent cations such as Nb and Ta to ZrO_2_ stabilized with trivalent cations such as Y and Yb increased fracture toughness [4]. Furthermore, the fracture toughness increased with the tetragonality (*c*/*a* ratio). If the *c*/*a* ratio is 1, the crystal phase is cubic. When pentavalent Nb and/or Ta are co-doped with Y and/or Yb, these cations stabilize the tetragonal structure and increase tetragonality by removing oxygen vacancies, forming charge compensating pairs such as Y–Nb, Yb–Nb, Y–Ta, and Yb–Ta [5,6,7,8]. Kim et al. demonstrated that the reduction in oxygen vacancies is responsible for the increased fracture toughness [5].

In addition to fracture toughness, thermal expansion increased with tetragonality [9,10]. Thermal expansion in the *c*-axis direction was greater than that in the *a*-axis direction across the entire composition range. This anisotropic thermal expansion behavior is attributed to the four-fold coordination of Nb^5+^ with oxygen ions in tetragonal ZrO_2_ solid solutions [11].

As mentioned above, while an increase in fracture toughness is desirable, an increase in the coefficient of thermal expansion (CTE) is not. To achieve the objective of increasing the fracture toughness without altering the CTE, Nb was applied solely to the surface. This approach resulted in an increase in fracture toughness for the entire specimen without a change in the CTE.

In this study, the IF method was employed to determine the depth profile of fracture toughness, as other methods such as the single-edge V-notch beam (SEVNB) and Chevron notch beam (CNB) are not suitable for small areas. The fracture toughness values for 3Y–TZP and 4.2Y–PSZ were determined to be 4.36 MPa√m and 3.61 MPa√m, respectively. These values differ from those reported in the literature [12,13,14,15] because the fracture toughness of zirconia varies depending on the measurement method and conditions. The effects of surface treatment on the fracture toughness of zirconia were evaluated using the same method and conditions to ensure consistency.

**Figure 11:**
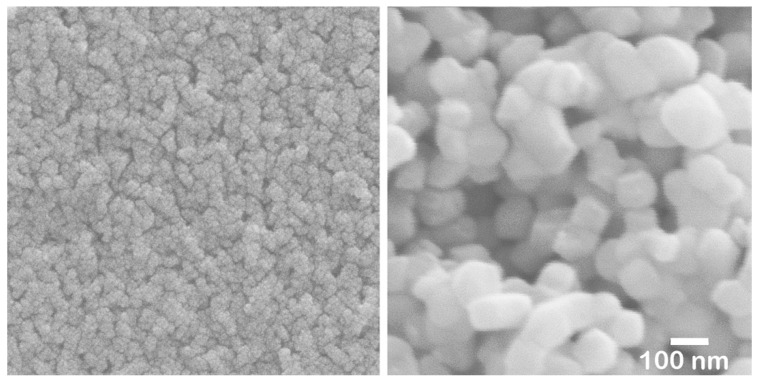
SEI of dried Nb sol (**left**) and pre-sintered body (**right**).

To selectively add Nb to the surface, a method involving the application or immersion of the surface in a compound containing Nb was considered. Although various compounds were tested, a high concentration of Nb could not be achieved on the surface using ionic solutions such as Nb chloride or Nb nitrate. The objective was only met when a Nb sol was applied or used for immersion. This sol is a water-based inorganic coating material composed of 6.0–6.4 wt% Nb_2_O_5_ nanoparticles, 0.2–0.7 wt% NH_3_, with the remainder being water. After drying at 60 °C for 16 h, nanosized Nb_2_O_5_ particles (less than 5 nm) agglomerated (Figure 11, left).

These nanoparticles in the sol are significantly smaller than the pore size of pre-sintered zirconia (Figure 11, right). Therefore, the sol can easily penetrate to a depth of approximately 1 mm from the surface but not deeper. After the final firing, a surface layer with high Nb content was formed to a depth of about 1 mm, as shown in Figure 5 and Figure 6. The concentration of surface-segregated Nb was equivalent to that of the bulk, which uniformly contained 1 mol% Nb_2_O_5_. Consequently, tetragonality increased only in the surface layer (Figure 9), and the fracture toughness also increased in this layer (Figure 10). This surface segregation was sufficient to enhance the fracture toughness of the entire body without increasing thermal expansion or decreasing translucency. The depth of segregation could be controlled by adjusting the sol concentration and the immersion time.

The effect of surface treatment on zirconia can be explained by the phenomenon caused by the formation of monoclinic crystals within tetragonal crystals. For example, sandblasting increases surface roughness, but the formation of monoclinic crystals generates compressive stress on the surface, improving bending strength [16,17]. On the other hand, it has been reported that when the amount of monoclinic crystal formed on the surface becomes too large due to low-temperature degradation, cracks occur and the strength decreases [17]. Thus, changes in the surface condition of zirconia affect the overall strength [18]. In this way, although the change is only on the surface, it convincingly results in an improvement in the overall strength. However, the CTE is independent of changes only on the surface; it is necessary to alter the material properties of the entire bulk.

The translucency of zirconia shows the same behavior. Our previous study demonstrated that the addition of Nb increases the opacity, i.e., reduces the translucency [4]. Again, when Nb is added only to the surface, the overall translucency is hardly affected. Therefore, the addition of pentavalent cations such as Nb, limited to the surface vicinity, improved the fracture toughness without affecting the CTE or the translucency.

Although the chemical stability of the Nb coating layer was not experimentally evaluated, it is considered fully stable based on previous studies [19,20,21]. According to these studies, a zirconia layer incorporating Nb is likely to be stable. ZrO_2_ containing Nb_2_O_5_, which forms Zr-Nb alloys, has been identified as a useful barrier to oxidation, indicating stability at high temperatures [19,20]. The introduction of Nb into tetragonal ZrO_2_ has been shown to enhance the concentration of Zr vacancies and of free electrons while reducing the concentration of oxygen vacancies [21]. Since water molecules can be incorporated into the ZrO_2_ lattice by filling oxygen vacancies [22], it is inferred that the Nb-incorporated zirconia layer is stable in water-based environments, such as those found in oral applications, due to the reduced oxygen vacancy concentration in this layer.

Even if it is made of zirconia, the joints of dental bridges where stress is likely to concentrate and the margins of dental crowns where the thickness is thin are prone to fracture. Adding Nb to the surface of the weak parts of zirconia prostheses can significantly enhance the overall strength and reliability of dental restorations. This approach leverages the beneficial properties of Nb addition to address the inherent weaknesses of zirconia, particularly at stress concentration points. Chemical durability studies and fatigue tests are necessary to evaluate the long-term stability of the treated layer. Additionally, the treatment method must be further refined to be practically applicable for fabricating zirconia prostheses.

## 5. Conclusions

The surfaces of pre-sintered 3Y–TZP and 4.2Y–PSZ were treated with a Nb sol solution containing Nb_2_O_5_ nanoparticles. After drying and final sintering, a surface layer with a high Nb content formed to a depth of approximately 1 mm. The concentration of surface-segregated Nb was equivalent to that of the bulk, which uniformly contained 1 mol% Nb_2_O_5_. The tetragonality of tetragonal zirconia in the surface vicinity increased with the addition of Nb, leading to improved fracture toughness in the surface layer and enhancing the fracture toughness of the entire specimen. However, the CTE and the translucency were independent of changes limited on the surface. Therefore, the addition of pentavalent cations such as Nb, confined to the surface vicinity, improved the fracture toughness without affecting the CTE or the translucency. By selectively adding Nb to the surface of the weak parts of zirconia prostheses, the overall strength can be improved, making it possible to produce highly reliable dental restorations. Further in vitro studies, combined with clinical investigations, are necessary to establish a practical technique for fabricating zirconia prostheses.

## Figures and Tables

**Table 1:** Chemical composition of specimens used in this study.

Code	Composition (mol%)		
	ZrO_2_	Y_2_O_3_	Nb_2_O_5_
3Y–TZP	97.0	3.0	-
4.2Y–PSZ	95.8	4.2	-
3Y–1 mol% Nb_2_O_5_	96.03	2.97	1.0
4.2Y–1 mol% Nb_2_O_5_	94.842	4.158	1.0

## Data Availability

The original contributions presented in the study are included in the article, further inquiries can be directed to the corresponding author.

## Abbreviations

CTE	coefficient of thermal expansion
TZP	tetragonal zirconia polycrystal
PSZ	partially stabilized zirconia
3Y–TZP	3 mol% yttria-stabilized tetragonal zirconia polycrystal
4Y–PSZ	4 mol% yttria-stabilized partially stabilized zirconia
4.2Y–PSZ	4.2 mol% yttria-stabilized partially stabilized zirconia
5Y–PSZ	5 mol% yttria-stabilized partially stabilized zirconia
UHTZ	ultra-high-translucent zirconia
CIP	cold isostatic press
IF method	indentation fracture method
SEVNB	single edge V-notch beam
CNB	chevron notch beam
SEM-WDX	scanning electron microscope with wavelength-dispersive X-ray spectroscopy
WDS PET	wave-dispersive X-ray spectroscopy using pentaerythritol analyzing crystal
XRD	X-ray diffraction
